# Novel age-dependent targets in vestibular schwannomas

**DOI:** 10.1186/1479-7364-8-10

**Published:** 2014-06-30

**Authors:** Amos Toren, Juergen K Reichardt, Ali Andalibi, Nancy Ya-Hsuan Hsu, Joni Doherty, William Slattery, Ruty Mehrian-Shai

**Affiliations:** 1 Department of Pediatric Hemato-Oncology, The Cancer Research Center, Sheba Medical Center, 2 Sheba Road, Ramat Gan 52621, Israel; 2 School of Pharmacy and Molecular Sciences, James Cook University, Townsville QLD 4811, Australia; 3 University of Connecticut, 438 Whitney Rd. Extension, Unit 1006, Storrs, CT 06269, USA; 4 Quest Diagnostics Nichols Institute, 14225 Newbrook Drive, Chantilly, VA 20153, USA; 5 Center for Neural Tumor Research, 2100 West Third Street, Los Angeles, CA 90057, USA; 6 Department of Clinical Studies, House Ear Institute, Los Angeles, CA 90057, USA; 7 Institute for Genetic Medicine, University of Southern California, Keck School of Medicine, Los Angeles, CA 90089, USA

**Keywords:** Neurofibromatosis, Schwannomas, Targeted therapy

## Abstract

**Background:**

Schwannomas are the most common neurofibromatosis type 2 (NF2)-associated tumors with significant phenotypic heterogeneity in patients. The most severe subtype has an early and rapid progression and the mild type has a later onset and a less aggressive course. The aim of this study was to elucidate the underlying molecular differences between these groups. We compared the gene expression pattern between patients with early to late age of onset.

**Results:**

A gene signature of 21 genes was constructed to differentiate between early-onset and late-onset patients. We confirmed these results by real-time PCR for SNF1LK2, NGFRAP1L1 (BEX 5), GMNN, and EPHA2.

**Conclusion:**

Genes identified here may be additional aberrations in merlin-depleted cells that govern the disease onset. A significant number of these genes have been suggested as having a role in carcinogenesis and are used as biomarkers for prognosis in several other cancers. The role of these genes in NF2 carcinogenesis and their potential as biomarkers or drug target are worthwhile exploring.

## Background

Neurofibromatosis type 2 (NF2) is an autosomal dominant disorder that predisposes affected individuals to several types of cerebral and spinal tumors [1]. Epidemiological studies place the incidence of NF2 at 1 in 25,000 live births [2]. The current treatment of NF2 is surgery and/or radiation therapy. However, there is no current safe therapeutic agent available at this time. Bilateral vestibular schwannomas (BVS) are the most common of NF2-associated tumors [3]. The consequences of a vestibular schwannomas (VS) are numerous, including dizziness, imbalance, tinnitus, hearing loss progressing to deafness, facial nerve paralysis, brainstem compression, and, if left untreated, death.

Mutations of the NF2 gene are thought to be the primary cause of this disease. Various types of mutations have been identified, among them single-base substitutions, insertions, and deletions [4]. The NF2 gene product, merlin, functions as a signal transduction pathway regulating cell-to-cell and cell matrix interactions. Merlin has been demonstrated to exert its antiproliferative effect by impact on several intracellular signaling pathways: inhibition of phosphatidylinositol 3-kinase (PI3-kinase), through binding to PI3-kinase enhancer, long form (PIKE-L) [5], modulation of the RAS/RAC oncogenic pathways [6], inhibition of the mitogen-activated protein kinase (MAPK) pathways [7] and repression of PAK-induced cyclin D1 expression [8]. Merlin regulates the MAPK signaling pathway by blocking the Cdc42-MLK3 interaction and MLK3 activity inhibition [9]. Furthermore, inactivation of merlin induces oncogenic gene expression, hyperproliferation, and tumorigenicity by releasing the inhibition of E3 ubiquitin ligase CRL4 (DCAF1) in the nucleus [10].

Since the disease has a variable presentation, with the severe subtype having an early and rapid progression and the milder type having a later onset and a less aggressive course, the diagnosis and management of these patients present a unique challenge [11]. Studies have indicated that a truncating NF2 mutation (nonsense and frameshift) is linked with the more severe form of NF2 [3]. The more severe form is particularly virulent, with unrelenting growth of schwannomas and meningiomas from childhood, resulting in blindness, deafness, paralysis, and death by the age of 40. The mild form of NF2 is less debilitating. The schwannomas may remain the same size for years, few meningiomas will develop. The patient may not develop symptoms until later in life and will have fewer disabilities [3].

There is considerable variability in growth rates of VS in patients with NF2, but they tend to be higher in patients who are younger at onset of signs or symptoms [12]. The most important predictors of disease severity are age at diagnosis and age at onset of symptoms. The average age of onset is 18 to 24 years. Almost all affected individuals develop bilateral vestibular schwannomas by age 30 years. Vestibular schwannomas are much less common in children than adults [13]. There are age-specific risks of NF2 to an offspring of a patient. Patient presenting with BVS <20 years 29.3% and patient presenting with asymmetric disease after 40 years 5.5% [14]. Patients of ages 35 years and below show higher p53 phosphorylation compared to the tumors of older patients [15].

The development of microarray tools assists effective, current, and comprehensive understanding of complex diseases including cancer [16]. Differences in gene expression between schwannomas or between schwannoma cells from NF2 patients and normal human primary schwann cells previously examined do not confer age impact [17-19]. Here, we report whole genome gene expression variation in NF tumors differing in age of onset. Improved understanding of these molecular differences may allow elucidation of age-dependent onset etiology, novel molecular markers for prognosis, and new therapeutic targets for neurofibromatosis.

## Results and discussion

**Figure 1:**
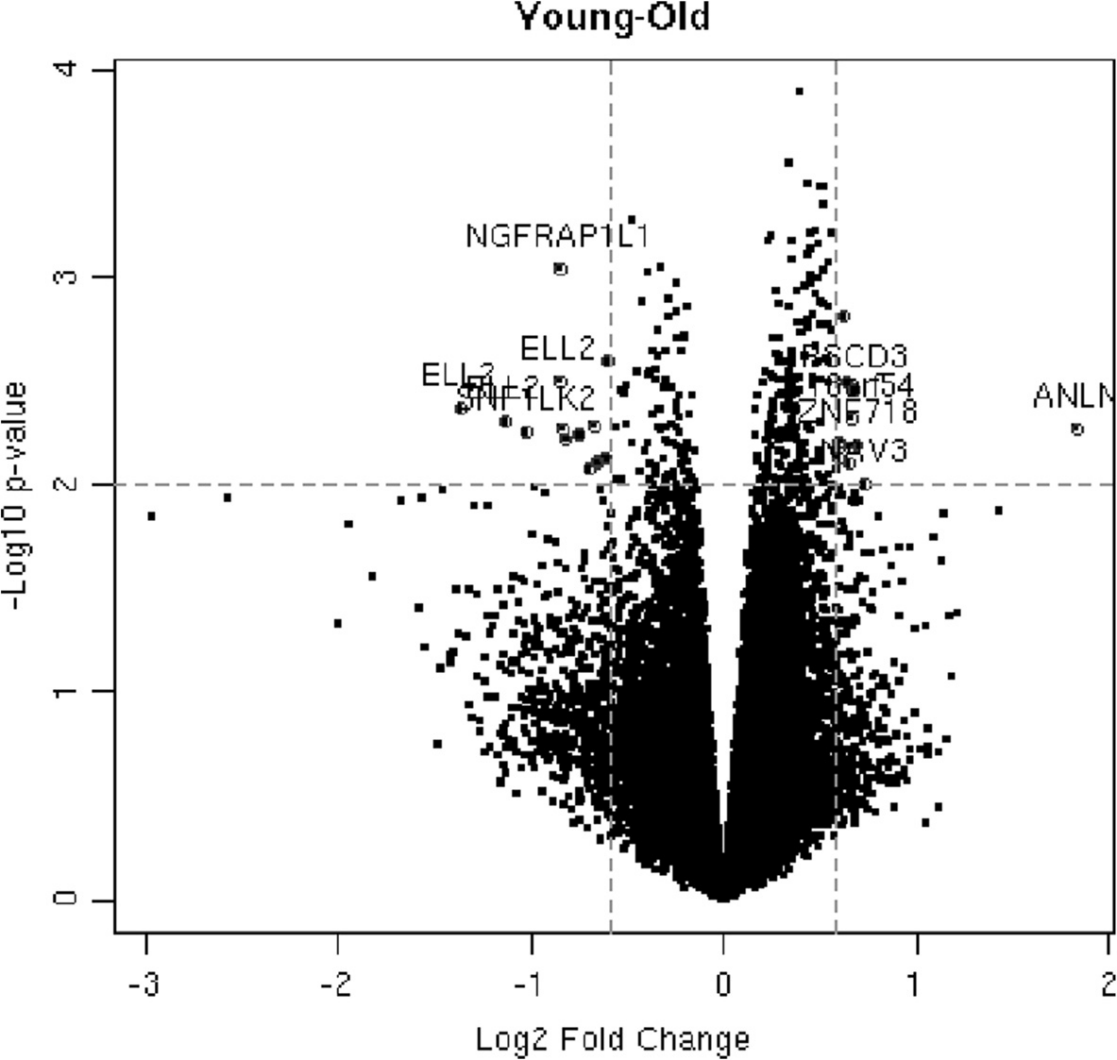
Volcano plot of early- and late-onset tumors.

**Figure 2:**
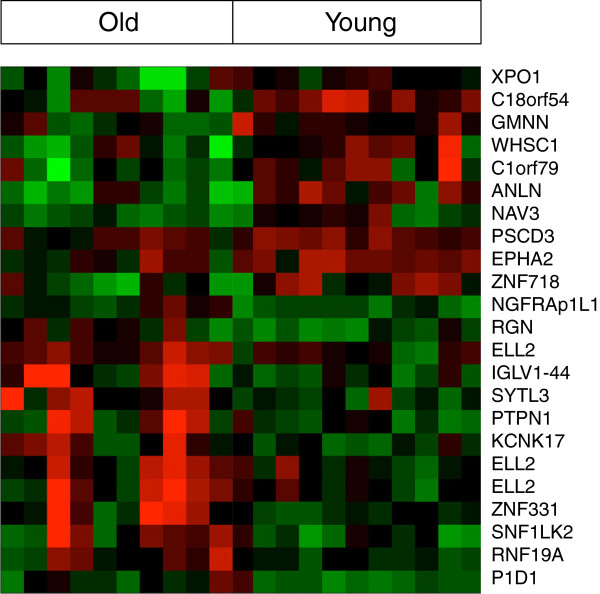
Heat map on age onset-dependent gene expression.

**Figure 3:**
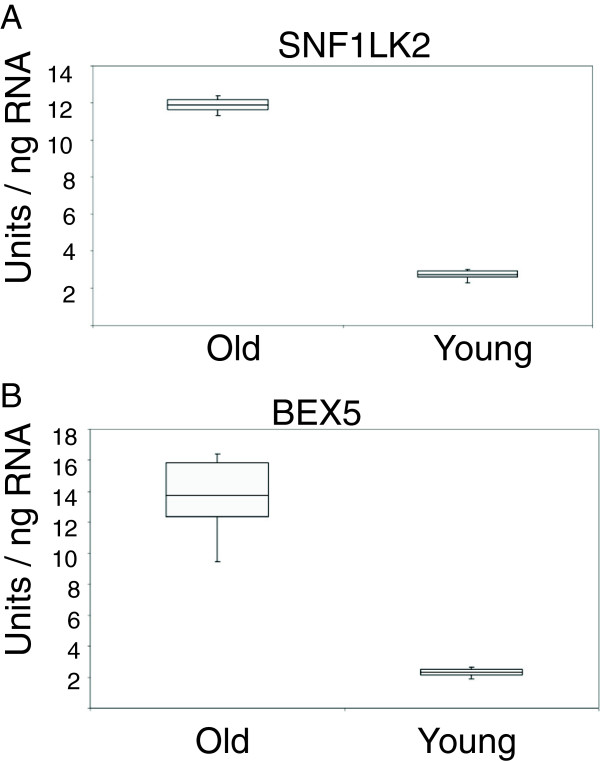
SNF1LK2 (A) and BEX5 (B) quantitative real-time PCR validation.

**Figure 4:**
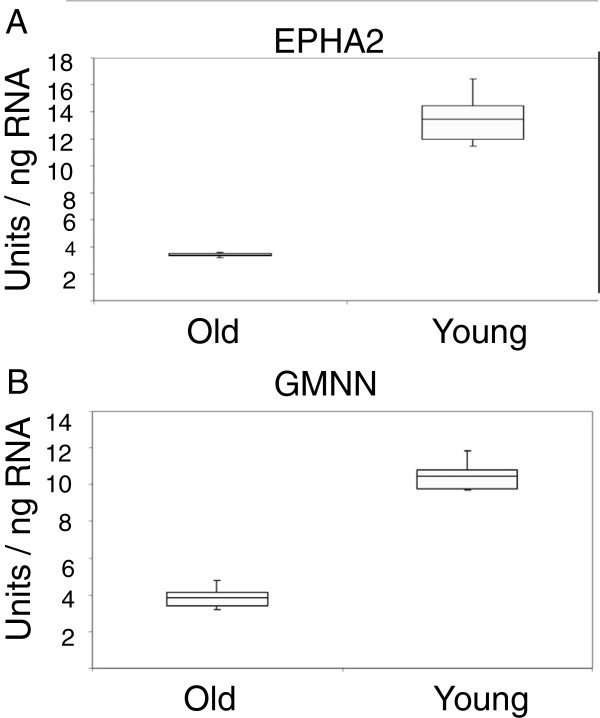
EPHA2 (A) and GMNN (B) quantitative real-time PCR validation.

We analyzed the transcriptome of VS samples from early-onset patients and late-onset patients. The results were summarized in a volcano scatter plot (Figure 1) that summarizes both fold change and *t* test criteria. The negative log_10_-transformed *p* values from the gene-specific *t* test was plotted against the log_2_ fold change between early and late onset. Genes with statistically significant differential expression according to the gene-specific *t* test lie above the horizontal threshold line. Genes with large fold change values lie outside the pair of vertical threshold lines. Twenty one significantly differentially expressed genes are identified by fold change >1.5 and *p* value <0.01: ANLN, NAV3, ZNF718, PSCD3, C180rf54, C10rf79, XPO1, GMNN, WHSC1, EPHA2, SNF1LK2, NGFRAP1L1, ELL2, SYTL3, KCNK17, IGLV1-44, PID1, RGN, PTPN1, ZNF331, and RNF19A (Table 1). Figure 2 illustrates the hierarchical expression of these genes for the two groups. The genes are clustered according to their relative expression in each group. Green color represents genes that have relatively low expression and red color represents genes that have relatively high expression. Finally, we utilized quantitative real-time RT-PCR to verify the results for four genes with the most potential future application according to the role they play in other cancers: SNF1LK2, NGFRAP1L1 (BEX 5), GMNN, and EPHA2 (Figures 3 and 4). In fact, RT-PCR confirmed the results for these genes and *t* test for all genes were significant at *p* value <0.005 (SNF1LK2 *p* value 2 × 10^-7^, NGFRAP1L1 (BEX5) *p* value 0.0002, GMNN *p* value 0.0009, and EPHA2 *p* value 9 × 10^-6^).

**Table 1:** List of age-dependent differentially expressed genes

**Gene symbol**	**Fold change**	***p *** **value**	**Function**	**Activity**	**Role in cancer**
ANLN	3.55	0.0053		Actin-binding protein	Overexpressed in pancreatic carcinoma
NAV3	1.65	0.0099	G	Neuron growth, regeneration	Neural tumorigenesis
ZNF718	1.61	0.0064		Zinc finger protein	Associated with prostate cancer
PSCD3	1.59	0.0035	T	Protein sorting and membrane trafficking	
C180rf54	1.57	0.0047			Overexpressed in multiple cancers
XPO1	1.56	0.0079	T/K	Nuclear protein transport export	
C10rf79	1.56	0.0065			
GMNN	1.55	0.0032	G	Proliferation marker	Correlated with poorer clinical outcome
WHSC1	1.51	0.006		Regulation of Wnt pathway	Expression increases with ascending glioma proliferation activity
EPHA2	1.51	0.0073	K	Tyrosine Kinase	Overexpressed in many types of cancer
SNF1LK2	-2.03	0.0055	K	Serine/Threonine kinase	Correlated with ovarian cancer poor survival
NGFRAP1L1 (BEX5)	-1.8	0.0009	G	Nerve growth factor receptor-associated	May be a tumor suppressor in VS
ELL2	-1.8	0.0031		Suppress canonical Wnt signaling	ELL translocations in malignancies.
SYTL3	-1.79	0.0053	T	Vesicular trafficking	
KCNK17	-1.76	0.006		Potassium channel	Expressed abnormally in many cancer types
IGLV1-44	-1.68	0.0057			
PID1	-1.62	0.0084		Glucose transport inhibitor	
RGN	-1.59	0.0052		Suppresses cell proliferation	Lost in breast and prostate cancer
PTPN1	-1.57	0.0076	K/G	Cell growth	
ZNF331	-1.53	0.0074	D	Induces apoptosis	
RNF19A	-1.52	0.0025	D	Neuronal cell death	

*T* transport, *K* kinase, *G* growth, *D* death.

Although the NF2 locus on chromosome 22 is associated with the formation of vestibular schwannomas, it is clear that in addition to merlin gene, there are a variety of other genes whose ‘dysregulation’ may eventually result in tumor formation. Comparison of gene expression between early- and late-onset specimens which all are merlin depleted provides a valuable insight into the modifications of these genes. Gene expression analysis of NF2 samples resulted in 21 genes that differentiate early-onset tumors from late-onset tumors (Table 1). Some of them are widely involved in nervous system activities and have been associated with cancer as described below and summarized in Table 2.

**Table 2:** Proposed applicable uses of differentially expressed genes

**Gene**	**Other cancer**	**Drug target**	**Outcome prediction**	**To be defined**
EPHA2	Y	Y	Y	
XPO1	Y	Y	Y	
SNF1LK2	Y	Y	Y	
NAV3	Y	Y	Y	
KCNK17	Y	Y	Y	
ZNF331	Y	Y	Y	
GMNN	Y		Y	
ZNF718	Y		Y	
WHSC1	Y			
ANLN	Y			
PSCD3	Y			
RGN	Y			
ELL2	Y			
PTPN1	Y			
BEX5				Y
SYTL3				Y
RNF19A				Y
P1D1				Y

Ten of the 21 genes we identified fall into four broad functional categories (Table 1): protein transport (genes PSCD3, XPO1, and SYTL3), protein phosphorylation and dephosphorylation (XPO1, EPHA2, and SNF1LK2), cell growth (NAV3, GMNN, and NGFRAP1L1), and cell death (ZNF331 and RNF19A). The latter two are particularly relevant to cancerous growth and the first two may underpin it as well. Additional cellular functions and cancer phenotypes are also listed in Table 1. Furthermore, some, at least 14 (EPA, XPO, SNF1LK, NAV3, KCNK17, ZNF331, GNMM, ZNF718, WHSC1, ANLN, PSCD3, RGN, ELL2, and PTPN1) of the 21 genes, have been reported to be involved in cancers (Table 1).

We further grouped the 21 genes we identified into 4 subgroups according to their potential role in NF2: in carcinogenesis, as biomarker, as drug target, or potential for clinical outcome prediction. This was according to other studies findings for each gene above. Another group was constructed from genes that their role in cancer has not been indicated yet (Table 2).

In summary, we report here a gene signature comprising 21 genes that characterize the clinically important age at onset in NF2 patients. Interestingly, many of these genes have known or at least inferred functions which may lead to a more complete molecular understanding of the age-based pathogenic differences in NF2 in the future. Such data may, in turn, lead to improved pre-symptomatic diagnoses and personalized age-focused treatment.

## Conclusion

There is great phenotypic difference between early and late onset in NF2. Our data indicate that these groups have distinct gene expression signatures. Most of the differentiating genes have already been shown to have important roles in other cancers. Thus, they may also have a role in VS NF2 patients. Moreover, these genes may be involved in pathogenesis, used as potential age-oriented biomarkers and treatment targets. Our gene lists will provide fertile ground for testing novel hypotheses on disease mechanism, pathogenesis, and therapeutic targets.

## Methods

Thirty-seven samples from human tumor (schwannomas) tissues were obtained at the time of surgery in accordance with applicable human ethics regulations (USC IRB protocol # 97–157) and snap frozen in liquid nitrogen. Tumor specimens were divided into two groups (young and old) according to the patient's age of onset. The young-onset group included 21 specimens from patients whose age of onset was less than 16 years old (ranging from 7 to 16 years old). The old-onset group included 16 specimens from patients whose age of onset was 30 years and older (ranging from 30 to 67 years old). The average age of the young group was 12 years old. The average age of the old group was 43 years. RNA was extracted and cRNA were hybridized to Human Genome U133 Plus 2.0 Array (Affymetrix) for analysis of over 47,000 transcripts. The detailed protocol for the sample preparation and microarray processing is available from Affymetrix at http://www.affymetrix.com (Santa Clara, CA, USA). For real-time PCR, total RNA was extracted and RT-qPCR was performed in the DNA Engine Opticon 2 Continuous Fluorescence Detection System (Bio-Rad, Hercules, CA, USA).

### Data analysis

The Bioconductor limma package [20] was used to preprocess the expression data from Human Genome U133 Plus 2.0 Affymetrix Array. To remove batch effects, the complete dataset were normalized by RMA. Before doing statistical analysis, we filter the probe sets by present/absent calls using the Wilcoxon signed rank-based algorithm. Out of 54,675 probes, 42,339 were kept and the remaining ones were removed as ‘Absent’ to reduce false positive rate. We identified differentially expressed gene list by fitting linear models to the normalized expression values. The empirical Bayes shrinkage was applied to *t* statistics using the limma package in Bioconductor. Because patient subtypes were still observed after RMA normalization, balanced samples from the same subtype were selected to identify significantly differentially expressed genes and to reduce the potential of small-sample artifacts. In general, selected genes have greater than 1.5 fold change and less than 0.01 *p* value.

The analysis of RT-PCR is as follows: Sample RNA was processed in triplicate with serial dilutions of Human Universal Reference RNA (Stratagene, La Jolla, CA, USA) on the same 96-well plate. Standard curves were constructed from the Universal RNA wells with arbitrary units of 1 unit equivalent to 1 pg of Universal RNA. Results were plotted as units per nanogram of RNA. Graphically, the middle line of the bar represents mean, bar size corresponds to the difference between lower quartile and upper quartile, and error bars represent the range of the raw data. *t* test *p* values were calculated for each differentiating gene.

### Ethical conduct of research

The authors state that they have obtained the University of Southern California institutional review board approval and have followed the principles outlined in the Declaration of Helsinki for all human experimental investigations. In addition, informed consent has been obtained from the participants involved.

## Competing interests

The authors declare that they have no competing interests.

## Authors’ contributions

AA and RMS developed the hypotheses underlying this study. JD, WS, and AA obtained informed consent and patient materials. AT had medical oversight of the study. RMS and NHYH performed the experimental procedures of this study. RMS, AT, and JKVR analyzed the data. RMS wrote the manuscript. AT and JKVR contributed to editing of the manuscript. All authors read and approved the final manuscript.
